# Association of concomitant continuous pain in trigeminal neuralgia with a narrow foramen ovale

**DOI:** 10.3389/fneur.2023.1277654

**Published:** 2023-10-31

**Authors:** Shuo Li, Chenlong Liao, Xiaosheng Yang, Wenchuan Zhang

**Affiliations:** Department of Neurosurgery, Shanghai Ninth People’s Hospital, Affiliated to Shanghai JiaoTong University School of Medicine, Shanghai, China

**Keywords:** trigeminal neuralgia, foramen ovale, concomitant continuous pain, etiology, atypical pain

## Abstract

**Background:**

The pathogenesis of concomitant continuous pain remains unclear and is worthy of further study. In this clinical study, we aimed to explore the potential role of a narrow foramen ovale in the development of concomitant continuous pain.

**Methods:**

A total of 108 patients with classical trigeminal neuralgia affecting the third branch of the trigeminal nerve and 46 healthy individuals were enrolled in this study. Three-dimensional reconstructed computerized tomography images of all participants were collected, and the morphometric features of the foramen ovale were examined by two investigators who were blinded to the clinical data of the patients.

**Results:**

In this cohort, patients with concomitant continuous pain suffered from more sensory abnormalities (18.4% vs. 2.9%, *p* = 0.015) and responded more poorly to medication (74.3% vs. 91.9%, *p* = 0.018) than patients without concomitant continuous pain. While no significant differences regarding the mean length (5.02 mm vs. 5.36 mm, *p* > 0.05) and area (22.14 mm^2^ vs. 23.80 mm^2^, *p* > 0.05) were observed between patients with and without concomitant continuous pain, the mean width of the foramen ovale on the affected side in patients with concomitant continuous pain was significantly narrower than that in patients without concomitant continuous pain (2.01 mm vs. 2.48 mm, *p* = 0.003).

**Conclusion:**

This neuroimaging and clinical study demonstrated that the development of concomitant continuous pain was caused by the compression of the trigeminal nerve owing to a narrow foramen ovale rather than responsible vessels in classical trigeminal neuralgia.

## Introduction

Trigeminal neuralgia (TN) is one of the most torturous pain conditions and is characterized by recurrent episodes of unbearable and electric-shock-like pain restricted to the territory of the trigeminal nerve (1, 2). In addition to the characteristic sharp pain, persistent background pain, described as throbbing and burning, between each attack has been reported to occur in 24%–49% of patients with TN (3–5). In 2018, the International Headache Society defined this condition as classical trigeminal neuralgia (CTN) with concomitant continuous pain (CCP), also termed atypical TN or TN type 2 (2, 6).

The pathogenesis of TN is complicated and has not been completely elucidated. To date, neurovascular compression (NVC) is the most convincing theory and is supported by the high rate of pain relief by microvascular decompression (MVD) (7–9). Nevertheless, the mechanisms giving rise to CCP are not fully understood, and even NVC can hardly explain the condition (10–12). Some researchers have hypothesized that the development of CCP may be associated with trigeminal nerve root atrophy, unmyelinated C fiber damage or central mechanisms, but substantive evidence is lacking (13–16). Although MVD is viewed as the most effective surgical procedure for TN (17, 18), a certain number of patients with persistent pain do not respond well to it (19–21). CCP is regarded as a major risk factor for poor outcomes for both medication treatment and MVD (22–29).

The foramen ovale (FO), through which the third branch of the trigeminal nerve (V3) and several small vessels travel, is an important bony aperture in the middle cranial fossa (30, 31). In recent years, several investigations have demonstrated that a narrow FO may be involved in the development of TN due to its potential to cause secondary persistent entrapment of V3 on the basis of NVC (32, 33). According to our prior clinical observations, patients with narrow FOs may present with persistent pain that is refractory to both medication and MVD. To further explore the mechanisms underlying CCP in TN, we measured and compared the anatomical characteristics of FOs among CTN patients with and without CCP in this clinical study.

## Materials and methods

### Patients

A total of 257 consecutive patients with TN diagnosed as TN according to ICHD-3 13.1.1 in our institute from January 2019 to January 2023 were screened in the study (2). Inclusion criteria were CTN with the involvement of the third branch of the trigeminal nerve (V3). Exclusion criteria were idiopathic TN, secondary TN, bilateral TN, skull base fracture and other orofacial pain. After screening, a total of 108 patients, consisting of 70 CTN patients without CCP and 38 CTN patients with CCP, were included. The pain type was determined prior to the first surgical treatments. Forty-six healthy controls (HCs), matched for sex and age, were also enrolled as the control group. HCs were those with head trauma who asked the doctors to order a CT scan to rule out intracranial hemorrhage and fractures. The demographic and clinical characteristics of all patients were recorded and are presented in Table 1.

**Table 1:** Demographic and clinical data in patients with and without concomitant continuous pain.

	CTN with CCP	CTN without CCP	*p* value
Total cases	38	70	
Age at onset (years)	65.5 ± 13.8	63.8 ± 9.0	0.459
Sex: female (*N*/%)	29(76.3%)	42(60.0%)	0.088
Side: right (*N*/%)	19(50.0%)	40(57.1%)	0.476
Distribution (*N*/%)			
V3	13 (34.2%)	24 (34/3%)	0.943
V2 + V3	21 (55.3%)	40 (57.1%)	
V1 + V2+ V3	4 (10.5%)	6(8.6%)	
Disease duration (months)	46.2 ± 38.8	59.9 ± 63.0	0.223
Response to drug (*N*/%)			
Yes	26 (68.4%)	57 (81.4%)	0.018^*^
No	9 (23.7%)	5 (7.15%)	
Other^#^	3 (7.9%)	8 (11.4%)	
Sensory abnormalities	7 (18.4%)	2 (2.9%)	0.015^*^

CTN: classical trigeminal neuralgia; CCP: concomitant continuous pain; *p < 0.05, ^#^drug allergy or no history of medication.

### Imaging examinations

**Figure 1:**
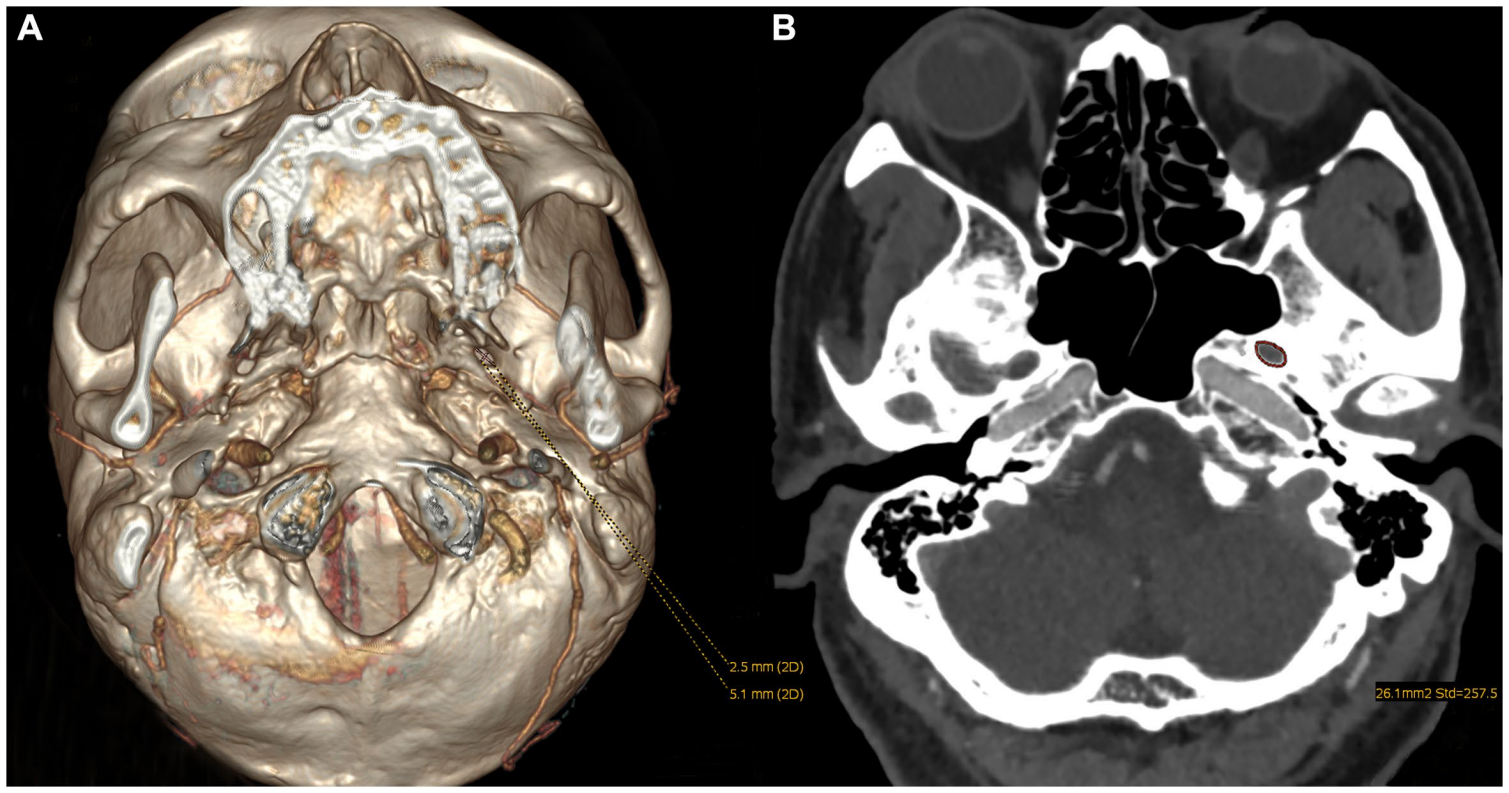
The parameters of an FO measured on CT images: **(A)** the width and length were measured on a 3D reconstruction image and **(B)** the area was measured on the axial image.

All obtained high-resolution head computed tomography (CT) images were transferred to the Picture Archiving and Communicating System and a 3-dimensional (3D) reconstruction was then created on a postprocessing workstation (Advantage Workstation 4.6, GE Healthcare, Milwaukee, WI). The width and length of each FO were measured on the 3D-CT image, and the area was measured on the corresponding axial CT image by a self-contained measuring tool (Figure 1). The details of the manual measurement method were stated in our previous article (34).

### Statistical analysis

Statistical analyses were performed using SPSS (Version 26; IBM Inc., New York). Continuous and categorical variables are presented as medians and numbers (percentages), respectively. An independent-sample T test or Wilcoxon signed-rank test was used for the comparison of continuous data, and a Chi-square test or Fisher exact test was used for the comparison of categorical data. A *p* value of <0.05 was defined as statistically significant. The study was reviewed and approved by our institutional review board.

## Results

### Patient characteristics

Patients with TN were divided into two groups according to the presence of CCP. The two patient groups did not differ in age (*p* = 0.459), sex (*p* = 0.088), side (*p* = 0.476), pain duration (*p* = 0.223) or distribution (*p* = 0.943). Age of disease onset was most commonly between 50 and 70 years, and pain tended to occur in female patients in both patient groups. In all patients in whom V3 was affected, the combination of V2 and V3 being affected was more frequent, followed by V3 alone. The distribution of CCP was consistent with the divisions of paroxysmal pain. Pain duration ranged from 1 month to 11 years (mean 3.9 years) in patients with CCP and from 1 month to 20 years (mean 5.0 years) in patients without CCP. Of the 38 patients with CCP, 26 (68.4%) had this continuous pain at disease onset; in the remaining 12 (31.6%) patients, CCP developed after a mean period of 2.1 years (range 6 months–10 years). Almost all patients with CCP described the pain as burning, dull, and not as severe as paroxysmal pain.

Conversely, the differences in response to medication and sensory abnormalities were statistically significant between groups. Owing to the impairment of the kidney and liver and other side effects, 3 patients with CCP and 8 patients without CCP did not receive the medication. The response to medication, such as carbamazepine, was poorer (*p* = 0.018) in patients with CCP. Although the response rate was high in both groups (91.9% vs. 74.3%) at onset, when visiting our center, the response rate decreased to 37.1% and 18.4%. Regarding sensory abnormalities, a small proportion of CTN patients developed abnormalities before surgery. Nevertheless, sensory abnormalities were much more prevalent in patients with CCP (18.4% vs. 2.9%, *p* = 0.015).

### FO characteristics

**Figure 2:**
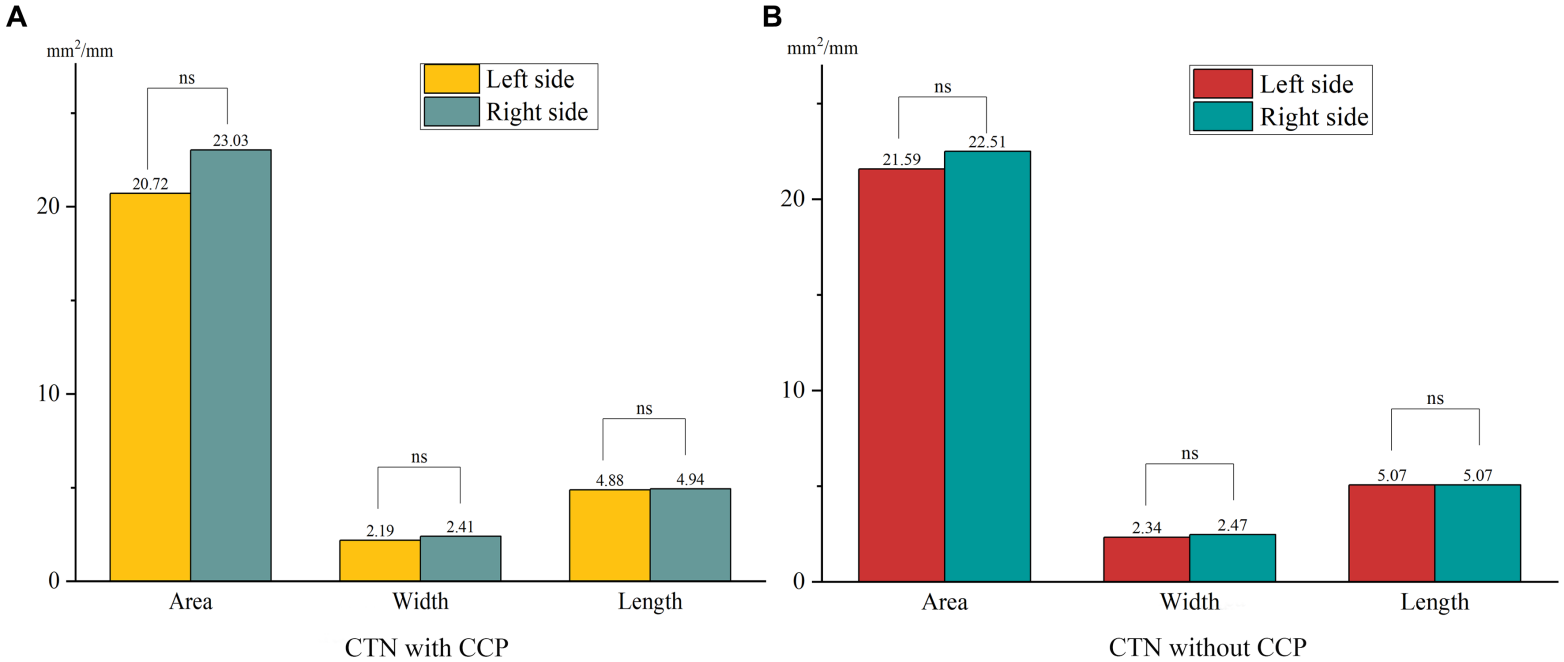
Differences in the size of FOs between the left and right sides in both patient groups. **(A)** No significant differences in the size of FOs between the left and right sides were observed in CTN patients with CCP. **(B)** No significant differences in the size of FOs between the left and right sides were observed in CTN patients without CCP. ns, no significant difference.

In the HC group, the average minimum area, width, and length of FOs on the left and right sides were 24.90 ± 7.13 mm^2^, 27.50 ± 10.39 mm^2^, 2.64 ± 0.69 mm, 2.77 ± 0.72 mm, 5.27 ± 1.17 mm, and 5.79 ± 1.33 mm, respectively. Although the length on the left side was significantly smaller than that on the right side, the difference in the area and width of FOs between the left and right sides was not statistically significant (*p* > 0.05). No significant differences in any parameters of FOs between the left and right sides in either patient group were found (Figure 2). The size of the FO did not correlate with age or sex, nor did it differ with disease duration.

**Figure 3:**
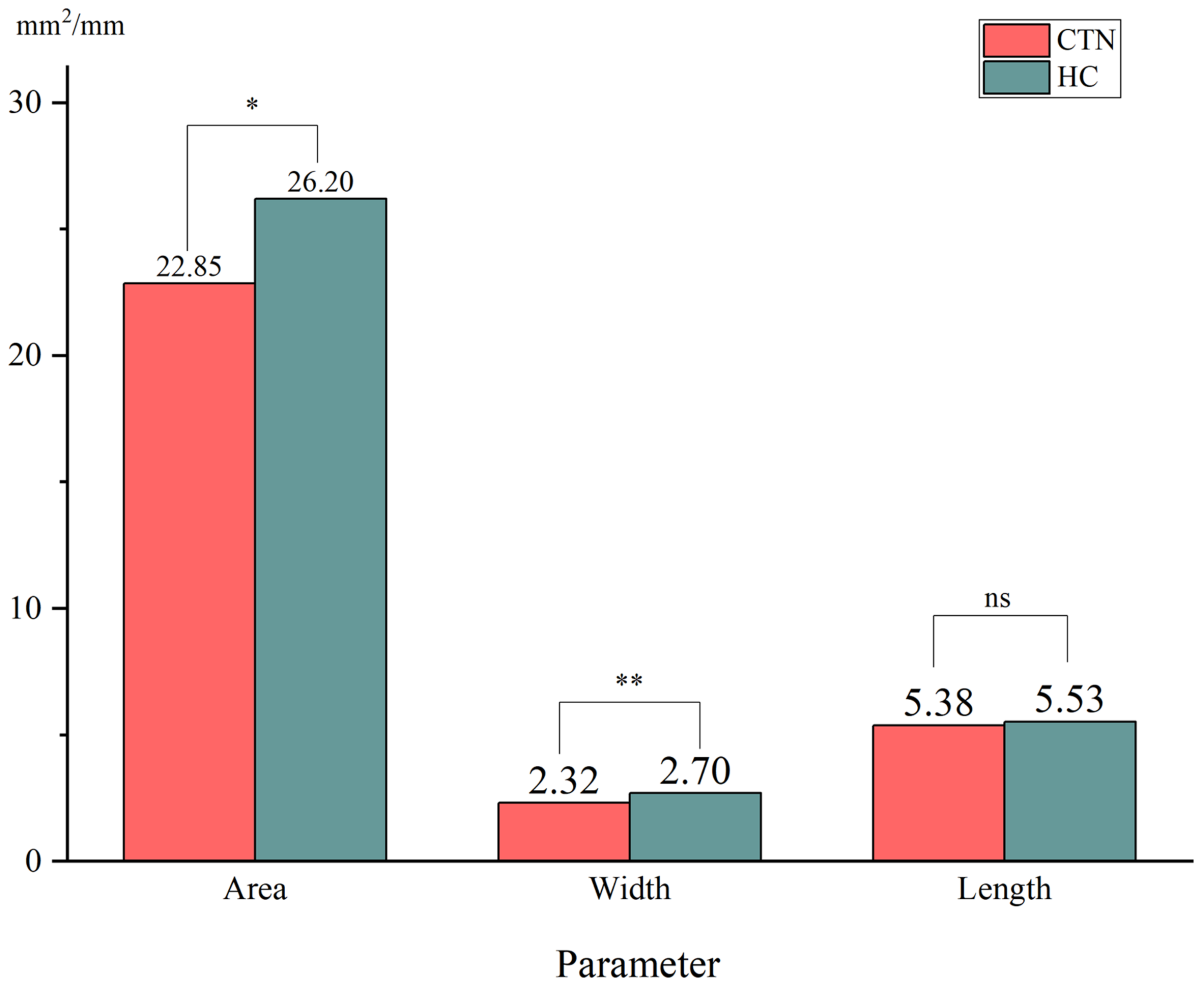
Differences in the size of FOs between the painful side in CTN patients and the painless side in healthy controls. ns, no significant difference. *p < 0.05, **p < 0.01.

**Figure 4:**
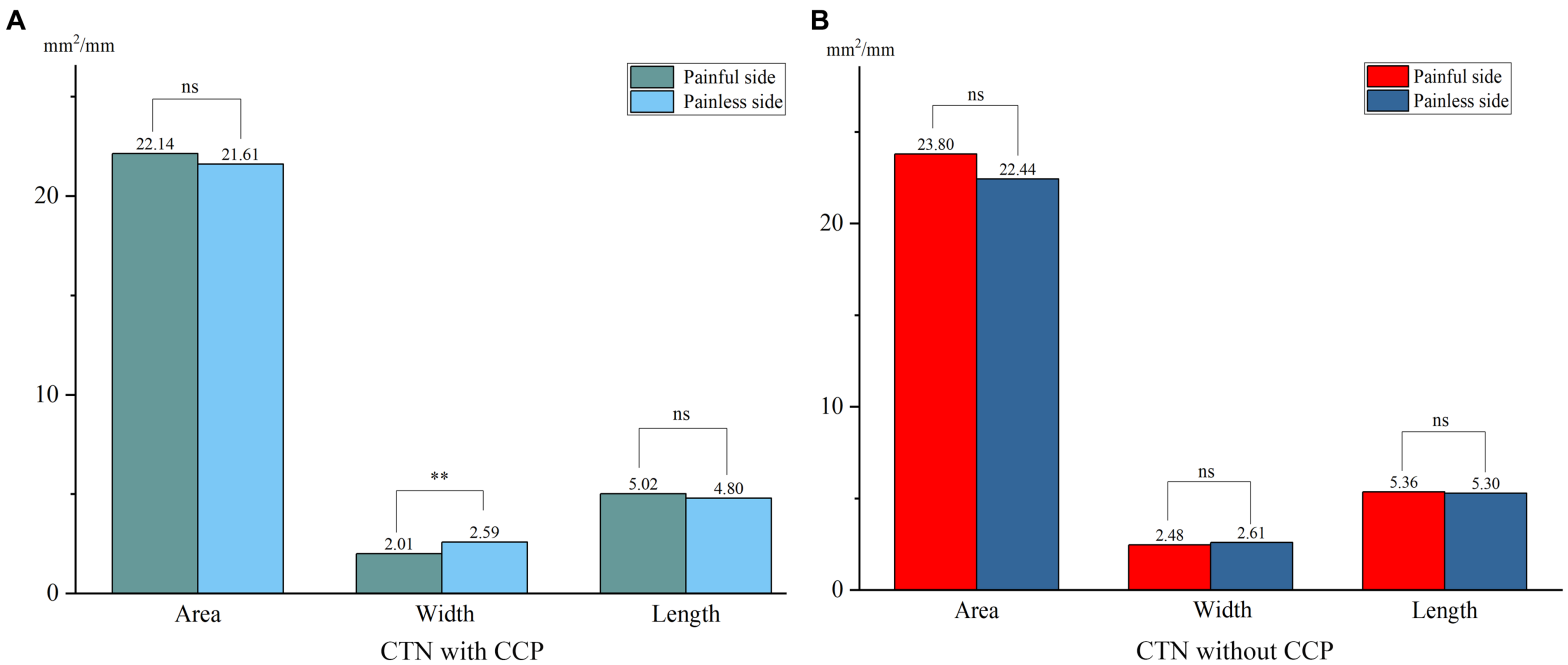
Differences in the size of FOs between the painful and painless sides in patients with or without CCP. **(A)** The width of FOs on the painless side was significantly smaller than that on the painless side, and no significant differences in area and length were observed in CTN patients with CCP. **(B)** No significant differences in the size of FOs between sides were observed in CTN patients without CCP. **p < 0.01, ns, no significant difference.

**Figure 5:**
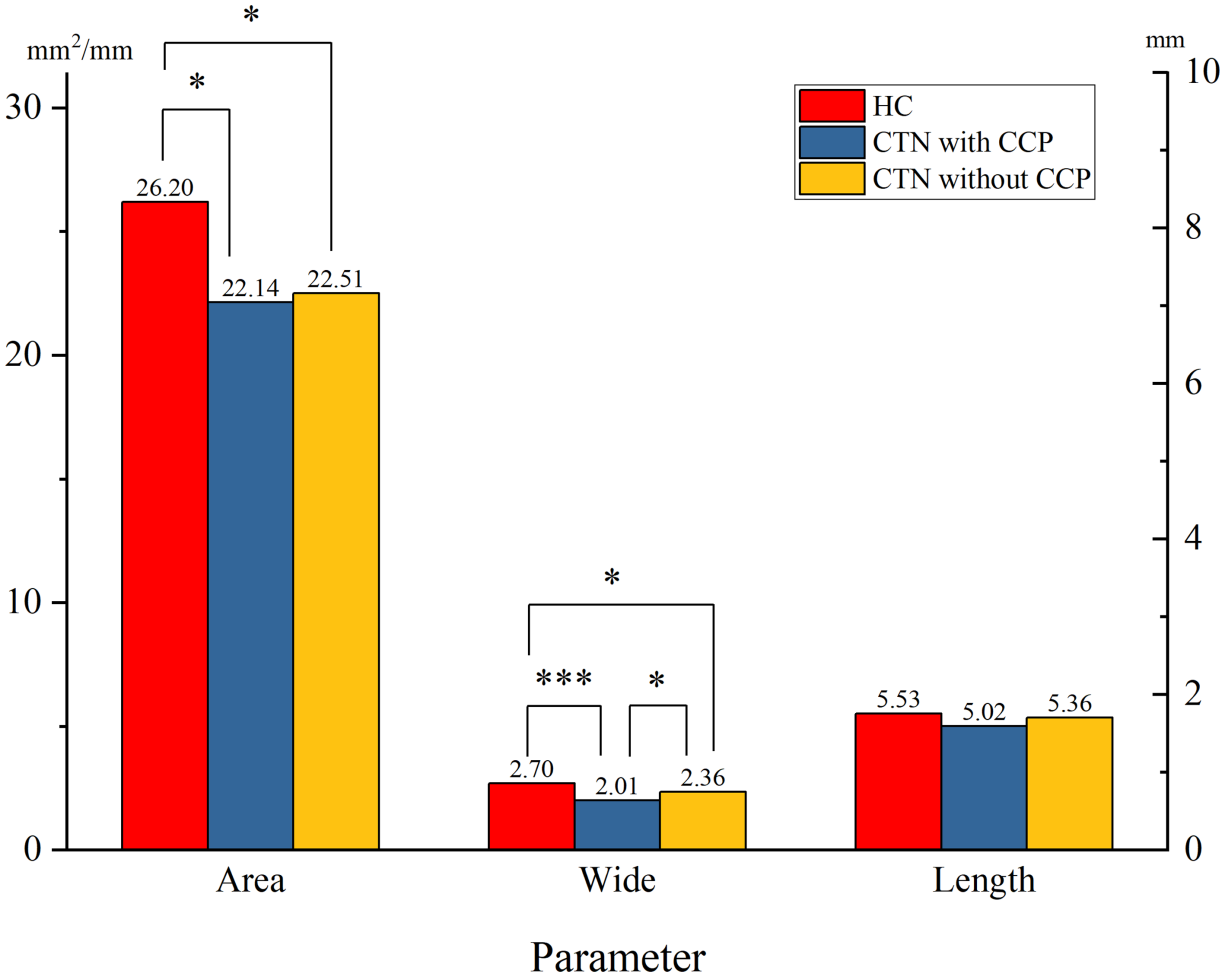
Differences in the size of FOs on the painful side between the two patient groups. ns, no significant difference; *p < 0.05, **p < 0.01, ***p < 0.001.

When comparing the patient group with the HC group, the mean width of the FO on the painful side in CTN patients was narrower than that in HCs (*p* = 0.005) (Figure 3). In patients with CCP, the mean area and width of FOs on the symptomatic side were significantly smaller than those of FOs on the asymptomatic side (*p* = 0.048, *p* < 0.001). In contrast, no difference in the size of FOs between the painful and painless sides in CTN patients without CCP was observed (*p* > 0.05) (Figure 4). Furthermore, the width of FOs on the painful side in patients with CCP was statistically narrower than that in patients without CCP (*p* = 0.003). Likewise, there were no significant differences in the area and length between the two patient groups (Figure 5).

## Discussion

With the deepening understanding of the pathogenesis of the characteristic paroxysmal pain, great strides in the treatment of TN have been made in recent decades. However, the underlying mechanisms causing CCP are still unknown. Furthermore, TN patients with CCP respond poorly to current available therapies, including pharmacological and surgical treatments (35–38). Therefore, clarifying the pathogenesis of CCP and finding effective therapies are urgently needed.

The hypothesis that a narrow FO compressing the trigeminal nerve secondarily could cause TN on the basis of the neurovascular compression theory was first proposed in 2005 by Neto et al. (33). Since then, relevant pathogenic mechanisms concerning the development of TN caused by a narrow FO have also been proposed (32, 39). Actually, it is not uncommon for peripheral nerves to be entrapped by narrow anatomical structures during their courses, and the double-crush theory has also been described in other diseases, such as painful diabetic peripheral neuropathy and carpal-tunnel syndromes (40–42). However, few studies have focused on the association between a narrow FO and TN, which may be because the characteristic pain in TN is paroxysmal rather than constant. In this clinical study, we enrolled 108 CTN patients and 46 HCs and analyzed their demographic and clinical data as well as FO characteristics retrospectively, aiming to explore whether a narrow FO is involved in the occurrence of CCP.

### Demographic and clinical characteristics

CCP occurred in 38 (35.2%) patients among the 108 CTN patients and was described as burning and throbbing but less intense than paroxysmal pain. The proportion and characteristics of CCP were similar to those reported in previous studies (4, 43). Approximately 68.4% of CCP cases developed at TN onset, but in 12 cases, CCP developed 6 months-10 years after paroxysmal pain. The differences in demographic data were not statistically significant. In this study, we found that CCP was equally distributed on the right (50%) and left sides (50%) in patients with CCP, which was distinct from previous conclusions (4). In contrast to patients without CCP, patients with CCP responded more poorly to medication (74.3% vs. 91.9%, *p* = 0.018). In addition, a small percentage of patients presented with sensory abnormalities in addition to pain. The uncomfortable sensation was much more prevalent in patients with CCP (18.4% vs. 2.9%, *p* = 0.015). Hitherto, no reasonable explanation accounts for the abovementioned phenomena.

### Characteristics of FOs

In this paper, we found no significant differences in the parameters of FOs between the left and right sides, suggesting that FOs exhibited bilateral symmetry. The average width and area of FOs on the affected side in CTN patients were significantly smaller than those on the painless side in HCs. The difference in the length was not significant. The results suggested that a narrow FO may be associated with the occurrence of CTN. Previous studies reached the same conclusion (34, 39). To further clarify the role played by a narrow FO in the development of CTN, the grouping method in this study was stricter, and patients were grouped into the CTN with CCP group and the CTN without CCP group according to the pain condition. There was no difference in FO width between CTN patients without CCP compared to HC cohort, however patients with CCP had narrower FO than healthy counterparts. Moreover, the width of the FOs on the painful side in patients with CCP was significantly narrower than that in patients without CCP. No significant difference in the area or length was observed. The above measurements and analysis indicated that a narrow FO compressing V3 constantly may contribute to the occurrence of CCP instead of paroxysmal pain.

Previous clinical studies could also provide some evidence to support our hypothesis. First, it has been demonstrated that sensory abnormalities and abnormal trigeminal reflex testing were more prevalent in patients with CCP than in patients without CCP, suggesting that the trigeminal nerve of patients with CCP may be damaged by factors other than NVC (43, 44). The characteristics of CCP are similar to the symptoms of nerve entrapment syndrome, which generally manifests as constant pain and sensory abnormalities rather than intermittent pain (40). The long-term entrapment of the trigeminal nerve by a narrow FO could give rise to trigeminal neuropathic pain. CTN with CCP may be the combination of TN with trigeminal neuropathic pain. Second, in a large proportion of CTN patients, paroxysmal pain was relieved after MVD; however, there was still persistent residual pain (45). The paroxysmal pain caused by responsible vessels can be resolved by MVD, while the entrapment due to a narrow FO cannot be resolved; therefore, CCP is still felt. Our opinion is supported by the findings of previous clinical studies suggesting that patients with CCP have poorer outcomes following MVD than CTN patients (38, 46). The phenomenon that patients with CCP respond poorly to sodium-channel blockers can also be explained by our hypothesis. Third, a number of TN patients present with CCP several months or years after the onset of paroxysmal pain (4, 36). This phenomenon is difficult to explain by existing mechanisms but contributes to our hypothesis. In our theory, with the accumulation of abnormal collagen and axonal contents, the trigeminal nerve presents progressive enlargement and swelling, which result in secondary entrapment of V3 in a narrow FO (47–50). Fourth, the second and third divisions of the trigeminal nerve are the most commonly affected in patients with CCP (4). Conversely, CCP seldom occurs in the distribution of the ophthalmic branch. The difference in the incidence of CCP at various divisions may be because the mandibular and maxillary branches are more likely to be compressed by a narrow FO and foramen rotundum during their courses. In addition, no difference in the incidence of CCP between the left and right sides was observed. Our measurement results demonstrating that FOs are bilaterally symmetrical support this phenomenon. Finally, recent studies have suggested that C fiber damage and axonal loss, which were also shown in other neuropathic pain conditions, may result in the development of CCP (51). However, it is still unclear how C fiber damage and axonal loss occur in TN with CCP. These could be attributed to the entrapment of the trigeminal nerve by a narrow FO, therefore resulting in burning, throbbing, or aching pain.

Our findings on the role of a double crush mechanism in CCP may have treatment implications. The optimum surgical treatment for CCP is still an open issue. On the basis of the aforementioned results, we suggest that it is necessary for surgeons to measure the parameters of the FOs on the symptomatic side for patients with CCP. Percutaneous procedures and gamma-knife radiosurgery may be considered the first choices for patients with a narrow FO. Future randomized controlled trials are needed to explore treatment strategies for achieving long-term pain relief in CTN patients with CCP.

## Limitations

Our study has several limitations. First, the retrospective design may affect the generalizability of results. Second, all parameters were measured manually and volume effects may lead to variable measurements. Finally, this study is hypothesis generating and need further validation by pathological and clinical studies.

## Conclusion

The findings of our clinical and neuroimaging study suggested that the width of FOs on the painful side in patients with CCP was significantly narrower, which demonstrated that a narrow FO may be involved in the occurrence of CCP and that the double crush theory may be the main mechanism of CCP. This paper may eventually help neurosurgeons select the optimum way to treat continuous pain in CTN patients.

## Data availability statement

The original contributions presented in the study are included in the article/supplementary material, further inquiries can be directed to the corresponding author.

## Ethics statement

The studies involving humans were approved by Shanghai Ninth People’s Hospital, Shanghai Jiao Tong University School of Medicine Ethics Committee. The studies were conducted in accordance with the local legislation and institutional requirements. The ethics committee/institutional review board waived the requirement of written informed consent for participation from the participants or the participants’ legal guardians/next of kin because the nature of this study is retrospective.

## Author contributions

SL: Writing – original draft. CL: Data curation, Investigation, Methodology, Writing – review & editing. XY: Resources, Software, Supervision, Writing – review & editing. WZ: Resources, Supervision, Validation, Visualization, Writing – review & editing.
